# Methyl 5-(2-bromo­acet­yl)-2-propoxybenzoate

**DOI:** 10.1107/S1600536808016814

**Published:** 2008-06-07

**Authors:** Jiang Ke, Xu Guan-Hong, Li Fei

**Affiliations:** a School of Pharmaceutical Science, Nanjing Medical University, Nanjing 210029, People’s Republic of China

## Abstract

The title compound, C_13_H_15_BrO_4_, was synthesized from methyl 5-acetyl-2-hydroxy­benzoate. With the exception of the ester group and some H atoms, the molecule is planar, the average deviation from planarity being 0.086 (5) Å. The dihedral angle between the phenyl ring and the ester group is 41.6 (3)°. Adjacent mol­ecules are inter­connected by C—H⋯O bonds, generating a layered structure.

## Related literature

For related literature, see: Grisar *et al.* (1981 ▶); Gronnow *et al.* (2005 ▶); Watanabe *et al.* (1984 ▶).
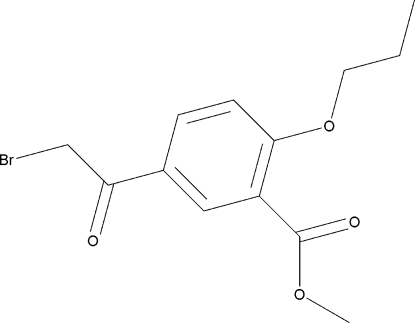


## Experimental

### 

#### Crystal data

C_13_H_15_BrO_4_
*M* *_r_* = 315.16
Monoclinic, 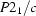

*a* = 16.292 (3) Å
*b* = 10.534 (2) Å
*c* = 7.8160 (16) Å
β = 92.42 (3)°
*V* = 1340.2 (5) Å^3^
*Z* = 4
Mo *K*α radiation
μ = 3.07 mm^−1^
*T* = 293 (2) K
0.30 × 0.10 × 0.09 mm

#### Data collection

Enraf–Nonius CAD-4 diffractometer
Absorption correction: ψ scan(North *et al.*, 1986 ▶) *T*_min_ = 0.459, *T*_max_ = 0.770
5055 measured reflections
2418 independent reflections
1255 reflections with *I* > 2σ(*I*)
*R*_int_ = 0.038
3 standard reflections every 200 reflections intensity decay: none

#### Refinement

*R*[*F* ^2^ > 2σ(*F* ^2^)] = 0.067
*wR*(*F* ^2^) = 0.163
*S* = 1.01
2418 reflections
163 parameters
H-atom parameters constrained
Δρ_max_ = 0.58 e Å^−3^
Δρ_min_ = −0.57 e Å^−3^

### 

Data collection: *CAD-4 Software* (Enraf–Nonius, 1989 ▶); cell refinement: *CAD-4 Software*; data reduction: *XCAD4* (Harms & Wocadlo, 1995 ▶); program(s) used to solve structure: *SHELXS97* (Sheldrick, 2008 ▶); program(s) used to refine structure: *SHELXL97* (Sheldrick, 2008 ▶); molecular graphics: *SHELXTL* (Sheldrick, 2008 ▶); software used to prepare material for publication: *SHELXTL*.

## Supplementary Material

Crystal structure: contains datablocks I, global. DOI: 10.1107/S1600536808016814/kp2172sup1.cif

Structure factors: contains datablocks I. DOI: 10.1107/S1600536808016814/kp2172Isup2.hkl

Additional supplementary materials:  crystallographic information; 3D view; checkCIF report

## Figures and Tables

**Table 1:** Hydrogen-bond geometry (Å, °)

*D*—H⋯*A*	*D*—H	H⋯*A*	*D*⋯*A*	*D*—H⋯*A*
C1—H1*A*⋯O1^i^	0.97	2.35	3.148 (9)	139
C8—H8*A*⋯O1^i^	0.93	2.59	3.500 (7)	165
C9—H9*A*⋯O2^ii^	0.96	2.61	3.534 (8)	162
C9—H9*C*⋯O3^iii^	0.96	2.59	3.501 (8)	158
C13—H13*A*⋯O2^iv^	0.97	2.59	3.484 (7)	154

Symmetry codes: (i) 
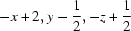
; (ii) 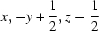; (iii) 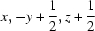; (iv) 
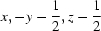
.

**Fig. 1.**
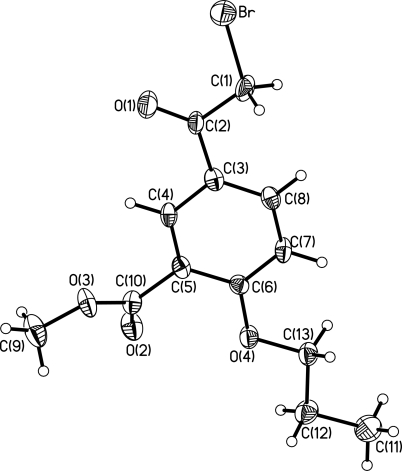
The molecular structure of (I), with atom labels and the 30% probability displacement ellipsoids for non-H atoms.

**Fig. 2.**
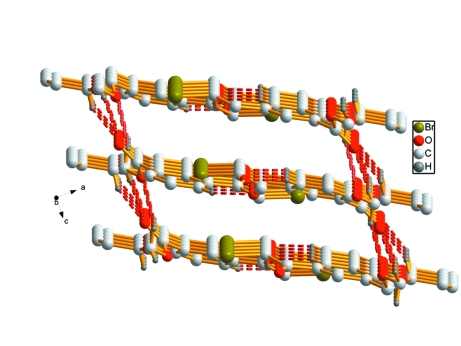
Layered structure generated by weak C—H···O hydrogen bonds .

## supplementary crystallographic information

### Comment

Methyl 5-acetyl-2-hydroxybenzoate is a common chemical intermediate, which can be easily obtained (Gronnow *et al.*, 2005). It is widely used for the design and synthesis of biological compounds. Biological activities, such as antiulcer (Watanabe *et al.*, 1984) and antihypertensive (Grisar *et al.*, 1981) effects of methyl 5-acetyl-2-hydroxybenzoate derivatives have been reported. In our research, the title compound, (I) (Fig. 1) is an important intermediate used to synthesize variety of compounds, which might have an  inhibitive effect on PDE5. Considerable attention has been devoted to the biological activities of  methyl 5-acetyl-2-hydroxybenzoate derivatives, however, the crystal structure of the title compound has not been reported, yet. In this work, we present the crystal structure of the title compound.

The molecule is planar with the average deviation from the planarity of 0.086 (5) Å. However, the ester group is out of this plane. The dihedral angle between the phenyl and the ester  group is 41.65°.

Packing analysis of the crystal structure shows that  molecules are intercontacted by weak C—H···O interactions generating a layered structure  (Table 1, Fig. 2).

### Experimental

Methyl 5-acetyl-2-propoxybenzoate was obtained by the alkylation of  methyl 5-acetyl-2-hydroxybenzoate. To a mixture of methyl 5-acetyl-2-propoxybenzoate (1 mmol), aluminium trichloride (0.15 mmol) and dichlormethane (15 mL), bromine (1.1 mmol) was added dropwise during 15 min at 273 K. The mixture was stirred at room temperature for 10 h. The resulting mixture was washed by aqueous solution of sodium thiosulfate, saturated salt solution, dried by anhydrous sodium sulfate, then the solvent was distilled off. Single crystals suitable for X-ray analysis (m.p. 379 K) were obtained by slow evaporation of solvent mixture of dichlormethane and methanol at room temperature.

### Refinement

H atoms were positioned geometrically and refined as riding atoms, with C—H = 0.96Å and *U*_iso_(H) = 1.5*U*_eq_(C) for methyl H atoms, 0.97Å and *U*_iso_(H) = 1.2*U*_eq_(C) for methylene H atoms, and C—H = 0.93Å and *U*_iso_(H) = 1.2*U*_eq_(C) for all other H atoms.

### Crystal data

**Table:**

C_13_H_15_BrO_4_	*F*_000_ = 640
*M**_r_* = 315.16	*D*_x_ = 1.562 Mg m^−^^3^
Monoclinic, *P*2_1_/*c*	Mo *K*α radiation λ = 0.71073 Å
*a* = 16.292 (3) Å	Cell parameters from 25 reflections
*b* = 10.534 (2) Å	θ = 10–13º
*c* = 7.8160 (16) Å	µ = 3.07 mm^−^^1^
β = 92.42 (3)º	*T* = 293 (2) K
*V* = 1340.2 (5) Å^3^	Block, colourless
*Z* = 4	0.30 × 0.10 × 0.09 mm

### Data collection

**Table:**

Enraf–Nonius CAD-4 diffractometer	*R*_int_ = 0.038
Radiation source: fine-focus sealed tube	θ_max_ = 25.2º
Monochromator: graphite	θ_min_ = 1.3º
*T* = 293(2) K	*h* = −19→19
ω/2θ scans	*k* = 0→12
Absorption correction: ψ scan(North et al., 1968)	*l* = 0→9
*T*_min_ = 0.459, *T*_max_ = 0.770	3 standard reflections
5055 measured reflections	every 200 reflections
2418 independent reflections	intensity decay: none
1255 reflections with *I* > 2σ(*I*)	

### Refinement

**Table:**

Refinement on *F*^2^	Secondary atom site location: difference Fourier map
Least-squares matrix: full	Hydrogen site location: inferred from neighbouring sites
*R*[*F*^2^ > 2σ(*F*^2^)] = 0.067	H-atom parameters constrained
*wR*(*F*^2^) = 0.163	*w* = 1/[σ^2^(*F*_o_^2^) + (0.070*P*)^2^ + 0.6*P*] where *P* = (*F*_o_^2^ + 2*F*_c_^2^)/3
*S* = 1.01	(Δ/σ)_max_ < 0.001
2418 reflections	Δρ_max_ = 0.59 e Å^−^^3^
163 parameters	Δρ_min_ = −0.57 e Å^−^^3^
Primary atom site location: structure-invariant direct methods	Extinction correction: none

### Special details

**Table:**

Geometry. All e.s.d.'s (except the e.s.d. in the dihedral angle between two l.s. planes) are estimated using the full covariance matrix. The cell e.s.d.'s are taken into account individually in the estimation of e.s.d.'s in distances, angles and torsion angles; correlations between e.s.d.'s in cell parameters are only used when they are defined by crystal symmetry. An approximate (isotropic) treatment of cell e.s.d.'s is used for estimating e.s.d.'s involving l.s. planes.
Refinement. Refinement of *F*^2^ against ALL reflections. The weighted *R*-factor *wR* and goodness of fit *S* are based on *F*^2^, conventional *R*-factors *R* are based on *F*, with *F* set to zero for negative *F*^2^. The threshold expression of *F*^2^ > σ(*F*^2^) is used only for calculating *R*-factors(gt) *etc*. and is not relevant to the choice of reflections for refinement. *R*-factors based on *F*^2^ are statistically about twice as large as those based on *F*, and *R*- factors based on ALL data will be even larger.

### Fractional atomic coordinates and isotropic or equivalent isotropic displacement parameters (Å^2^)

**Table:**

	*x*	*y*	*z*	*U*_iso_*/*U*_eq_	
Br	0.87726 (4)	−0.11114 (8)	0.09429 (10)	0.0697 (3)	
O1	1.0062 (3)	0.0376 (4)	0.2942 (6)	0.0686 (13)	
C1	0.9832 (4)	−0.1652 (7)	0.1690 (10)	0.067 (2)	
H1A	0.9781	−0.2417	0.2368	0.080*	
H1B	1.0141	−0.1867	0.0696	0.080*	
O2	1.3189 (3)	0.0316 (4)	0.7514 (4)	0.0528 (11)	
C2	1.0319 (3)	−0.0665 (5)	0.2757 (7)	0.0404 (13)	
O3	1.2859 (3)	0.1509 (4)	0.5226 (5)	0.0500 (11)	
C3	1.1118 (3)	−0.1133 (5)	0.3539 (6)	0.0358 (12)	
O4	1.3340 (2)	−0.2111 (3)	0.5971 (4)	0.0428 (9)	
C4	1.1620 (3)	−0.0215 (5)	0.4371 (6)	0.0362 (12)	
H4A	1.1452	0.0630	0.4379	0.043*	
C5	1.2358 (3)	−0.0556 (5)	0.5178 (6)	0.0346 (12)	
C6	1.2604 (3)	−0.1825 (5)	0.5180 (6)	0.0348 (12)	
C7	1.2117 (3)	−0.2720 (5)	0.4331 (6)	0.0420 (13)	
H7A	1.2287	−0.3562	0.4305	0.050*	
C8	1.1383 (3)	−0.2374 (6)	0.3525 (6)	0.0397 (13)	
H8A	1.1062	−0.2988	0.2963	0.048*	
C9	1.3255 (5)	0.2576 (7)	0.6034 (8)	0.069 (2)	
H9A	1.3221	0.3293	0.5277	0.104*	
H9B	1.3821	0.2376	0.6295	0.104*	
H9C	1.2988	0.2774	0.7073	0.104*	
C10	1.2855 (3)	0.0437 (6)	0.6120 (7)	0.0399 (13)	
C11	1.4632 (4)	−0.4854 (7)	0.7446 (10)	0.074 (2)	
H11A	1.5150	−0.4884	0.8077	0.111*	
H11B	1.4674	−0.5307	0.6387	0.111*	
H11C	1.4216	−0.5240	0.8109	0.111*	
C12	1.4400 (4)	−0.3468 (6)	0.7070 (8)	0.0524 (16)	
H12A	1.4372	−0.3001	0.8135	0.063*	
H12B	1.4815	−0.3076	0.6390	0.063*	
C13	1.3581 (3)	−0.3422 (6)	0.6111 (7)	0.0423 (14)	
H13A	1.3623	−0.3791	0.4981	0.051*	
H13B	1.3176	−0.3899	0.6722	0.051*	

### Atomic displacement parameters (Å^2^)

**Table:**

	*U*^11^	*U*^22^	*U*^33^	*U*^12^	*U*^13^	*U*^23^
Br	0.0589 (4)	0.0640 (5)	0.0837 (6)	0.0063 (4)	−0.0256 (3)	−0.0101 (4)
O1	0.069 (3)	0.038 (3)	0.095 (3)	0.010 (3)	−0.035 (3)	−0.013 (3)
C1	0.063 (4)	0.043 (4)	0.092 (5)	0.017 (3)	−0.027 (4)	−0.038 (4)
O2	0.075 (3)	0.044 (2)	0.038 (2)	−0.007 (2)	−0.019 (2)	0.000 (2)
C2	0.048 (3)	0.023 (3)	0.050 (3)	0.003 (3)	−0.007 (3)	−0.001 (3)
O3	0.077 (3)	0.031 (2)	0.040 (2)	−0.016 (2)	−0.0199 (19)	0.0067 (19)
C3	0.049 (3)	0.036 (3)	0.022 (3)	−0.004 (3)	−0.002 (2)	−0.005 (2)
O4	0.050 (2)	0.032 (2)	0.045 (2)	0.0014 (18)	−0.0100 (17)	−0.0032 (18)
C4	0.054 (3)	0.027 (3)	0.028 (3)	−0.001 (3)	0.000 (2)	−0.007 (2)
C5	0.047 (3)	0.034 (3)	0.024 (3)	−0.006 (3)	0.005 (2)	−0.008 (2)
C6	0.035 (2)	0.035 (3)	0.033 (3)	−0.003 (2)	−0.004 (2)	−0.003 (2)
C7	0.059 (3)	0.024 (3)	0.042 (3)	0.002 (3)	−0.005 (3)	0.001 (3)
C8	0.049 (3)	0.036 (3)	0.034 (3)	−0.006 (3)	−0.002 (2)	−0.005 (3)
C9	0.099 (5)	0.047 (4)	0.059 (4)	−0.020 (4)	−0.025 (4)	0.004 (3)
C10	0.051 (3)	0.034 (3)	0.034 (3)	0.002 (3)	0.001 (3)	−0.001 (3)
C11	0.066 (4)	0.064 (5)	0.090 (5)	0.006 (4)	−0.018 (4)	0.014 (4)
C12	0.054 (3)	0.050 (4)	0.051 (3)	0.011 (3)	−0.014 (3)	−0.002 (3)
C13	0.050 (3)	0.034 (3)	0.042 (3)	0.001 (3)	−0.005 (3)	−0.005 (3)

### Geometric parameters (Å, °)

**Table:**

Br—C1	1.887 (6)	C6—C7	1.382 (7)
O1—C2	1.185 (7)	C7—C8	1.377 (7)
C1—C2	1.532 (8)	C7—H7A	0.9300
C1—H1A	0.9700	C8—H8A	0.9300
C1—H1B	0.9700	C9—H9A	0.9600
O2—C10	1.204 (6)	C9—H9B	0.9600
C2—C3	1.498 (7)	C9—H9C	0.9600
O3—C10	1.328 (7)	C11—C12	1.534 (10)
O3—C9	1.430 (7)	C11—H11A	0.9600
C3—C8	1.377 (8)	C11—H11B	0.9600
C3—C4	1.408 (7)	C11—H11C	0.9600
O4—C6	1.360 (6)	C12—C13	1.504 (7)
O4—C13	1.439 (7)	C12—H12A	0.9700
C4—C5	1.382 (7)	C12—H12B	0.9700
C4—H4A	0.9300	C13—H13A	0.9700
C5—C6	1.396 (8)	C13—H13B	0.9700
C5—C10	1.498 (8)		
			
C2—C1—Br	114.2 (4)	C7—C8—H8A	119.5
C2—C1—H1A	108.7	O3—C9—H9A	109.5
Br—C1—H1A	108.7	O3—C9—H9B	109.5
C2—C1—H1B	108.7	H9A—C9—H9B	109.5
Br—C1—H1B	108.7	O3—C9—H9C	109.5
H1A—C1—H1B	107.6	H9A—C9—H9C	109.5
O1—C2—C3	124.0 (5)	H9B—C9—H9C	109.5
O1—C2—C1	121.1 (5)	O2—C10—O3	123.7 (5)
C3—C2—C1	114.8 (5)	O2—C10—C5	125.8 (5)
C10—O3—C9	116.6 (4)	O3—C10—C5	110.5 (4)
C8—C3—C4	118.7 (5)	C12—C11—H11A	109.5
C8—C3—C2	125.2 (5)	C12—C11—H11B	109.5
C4—C3—C2	116.1 (5)	H11A—C11—H11B	109.5
C6—O4—C13	118.6 (4)	C12—C11—H11C	109.5
C5—C4—C3	120.6 (5)	H11A—C11—H11C	109.5
C5—C4—H4A	119.7	H11B—C11—H11C	109.5
C3—C4—H4A	119.7	C13—C12—C11	109.4 (6)
C4—C5—C6	119.6 (5)	C13—C12—H12A	109.8
C4—C5—C10	119.0 (5)	C11—C12—H12A	109.8
C6—C5—C10	121.3 (5)	C13—C12—H12B	109.8
O4—C6—C7	123.0 (5)	C11—C12—H12B	109.8
O4—C6—C5	117.4 (4)	H12A—C12—H12B	108.2
C7—C6—C5	119.6 (4)	O4—C13—C12	107.6 (5)
C8—C7—C6	120.6 (5)	O4—C13—H13A	110.2
C8—C7—H7A	119.7	C12—C13—H13A	110.2
C6—C7—H7A	119.7	O4—C13—H13B	110.2
C3—C8—C7	120.9 (5)	C12—C13—H13B	110.2
C3—C8—H8A	119.5	H13A—C13—H13B	108.5

### Hydrogen-bond geometry (Å, °)

**Table:**

*D*—H···*A*	*D*—H	H···*A*	*D*···*A*	*D*—H···*A*
C1—H1A···O1^i^	0.97	2.35	3.148 (9)	139
C8—H8A···O1^i^	0.93	2.59	3.500 (7)	165
C9—H9A···O2^ii^	0.96	2.61	3.534 (8)	162
C9—H9C···O3^iii^	0.96	2.59	3.501 (8)	158
C13—H13A···O2^iv^	0.97	2.59	3.484 (7)	154

Symmetry codes: (i) −*x*+2, *y*−1/2, −*z*+1/2; (ii) *x*, −*y*+1/2, *z*−1/2; (iii) *x*, −*y*+1/2, *z*+1/2; (iv) *x*, −*y*−1/2, *z*−1/2.

## References

[bb1] Enraf–Nonius (1989). *CAD-4 Software* Enraf–Nonius, Delft, The Netherlands.

[bb2] Grisar, J. M., Claxton, G. P., Bare, T. M., Dage, R. C., Cheng, H. C. & Woodward, J. K. (1981). *J. Med. Chem.* **24**, 327–336. 10.1021/jm00135a017 6115059

[bb3] Gronnow, M. J., White, R. J., Clark, J. H. & Macquarrie, D. J. (2005). *Org. Process Res. Dev.* **9**, 516–518.

[bb7] Harms, K. & Wocadlo, S. (1995). (2004). *XCAD4* University of Marburg, Germany.

[bb4] North, A. C. T., Phillips, D. C. & Mathews, F. S. (1968). *Acta Cryst.* A**24**, 351–359.

[bb5] Sheldrick, G. M. (2008). *Acta Cryst.* A**64**, 112–122. 10.1107/S0108767307043930 18156677

[bb6] Watanabe, M., Kawada, M., Takamoto, M., Imada, I. & Noguchi, S. (1984). *Chem. Pharm. Bull.* **32**, 3372–3377.

